# Image-Guided Surgery in Patients with Pancreatic Cancer: First Results of a Clinical Trial Using SGM-101, a Novel Carcinoembryonic Antigen-Targeting, Near-Infrared Fluorescent Agent

**DOI:** 10.1245/s10434-018-6655-7

**Published:** 2018-07-26

**Authors:** Charlotte E. S. Hoogstins, Leonora S. F. Boogerd, Babs G. Sibinga Mulder, J. Sven D. Mieog, Rutger Jan Swijnenburg, Cornelis J. H. van de Velde, Arantza Farina Sarasqueta, Bert A. Bonsing, Berenice Framery, André Pèlegrin, Marian Gutowski, Françoise Cailler, Jacobus Burggraaf, Alexander L. Vahrmeijer

**Affiliations:** 1 Department of Surgery, Leiden University Medical Center, Leiden, The Netherlands; 2 Centre for Human Drug Research, Leiden, The Netherlands; 3 Department of Pathology, Leiden University Medical Center, Leiden, The Netherlands; 4 Surgimab, Montpellier, France; 5 Institut de Recherche en Cancérologie de Montpellier, Montpellier, France; 6 Institut Régional du Cancer de Montpellier, Montpellier, France; 7 Leiden Academic Center for Drug Research, Leiden, The Netherlands

## Abstract

**Background:**

Near-infrared (NIR) fluorescence is a promising novel imaging technique that can aid in intraoperative demarcation of pancreatic cancer (PDAC) and thus increase radical resection rates. This study investigated SGM-101, a novel, fluorescent-labeled anti-carcinoembryonic antigen (CEA) antibody. The phase 1 study aimed to assess the tolerability and feasibility of intraoperative fluorescence tumor imaging using SGM-101 in patients undergoing a surgical exploration for PDAC.

**Methods:**

At least 48 h before undergoing surgery for PDAC, 12 patients were injected intravenously with 5, 7.5, or 10 mg of SGM-101. Tolerability assessments were performed at regular intervals after dosing. The surgical field was imaged using the Quest NIR imaging system. Concordance between fluorescence and tumor presence on histopathology was studied.

**Results:**

In this study, SGM-101 specifically accumulated in CEA-expressing primary tumors and peritoneal and liver metastases, allowing real-time intraoperative fluorescence imaging. The mean tumor-to-background ratio (TBR) was 1.6 for primary tumors and 1.7 for metastatic lesions. One false-positive lesion was detected (CEA-expressing intraductal papillary mucinous neoplasm). False-negativity was seen twice as a consequence of overlying blood or tissue that blocked the fluorescent signal.

**Conclusion:**

The use of a fluorescent-labeled anti-CEA antibody was safe and feasible for the intraoperative detection of both primary PDAC and metastases. These results warrant further research to determine the impact of this technique on clinical decision making and overall survival.

Surgical resection is the only possible curative treatment for patients with pancreatic ductal adenocarcinoma (PDAC). Differentiation between tumoral and non-tumoral tissue is often difficult during PDAC surgery, and this can lead to incomplete cancer removal, exemplified by high percentages of irradical resections.^1^–^3^ As survival time doubles for patients with a microscopically radical resection,^4^ enhanced intraoperative visualization of PDAC is crucial to increase radical resection rates and improve patient outcomes. Image-guided surgery using near-infrared (NIR) fluorescence is a novel imaging technique that can aid in real-time demarcation of tumors during surgery.^5^

To detect tumors using NIR fluorescence imaging, a fluorescent contrast agent is administered, and the distribution of the agent is visualized using a dedicated imaging system. At first, the fluorescence-imaging field focused predominantly on indocyanine green (ICG), a nonspecific imaging agent, because ICG is available for clinical use. Exploiting the hypothesized passive accumulation of ICG in tumor cells, a study in PDAC was performed.^6^ The results were unfavorable because sufficient contrast between the tumor and surrounding pancreatic tissue was not achieved.

Currently, the fluorescence-imaging field focuses mainly on tumor-specific agents (e.g., antibodies or ligands conjugated to a fluorophore) that molecularly target biomarkers expressed by tumor cells. Recently, clinical trials (including one randomized controlled trial) with these tumor-specific fluorescent agents in other cancer types have demonstrated feasibility and potential clinical benefits of image-guided surgery.^7^^–^9 However, tumor-specific agents for fluorescence imaging of PDAC have not been investigated.

A number of tumor-specific imaging agent seem to have high potential for specific fluorescence imaging of PDAC including integrin αvβ6, carcinoembryonic antigen (CEA), epithelial growth factor receptor (EGFR), and urokinase plasminogen activator receptor (uPAR).^10^ As a glycoprotein produced by gastrointestinal tissue, CEA is overexpressed in many cancer types, including pancreatic ductal adenocarcinoma.^11^,^12^ Antibodies directed toward CEA-overexpressing tumors for various therapeutic applications have been successfully tested in trials.^13^–^15^ Moreover, imaging studies using positron emission tomography (PET) and radiolabelled anti-CEA antibodies have demonstrated high tumor uptake and good contrast.^16^,^17^

This study investigated the use of a novel fluorescent-labeled (700 nm) anti-CEA antibody for clinical application. In PDAC orthotopic mouse models using BxPC3 cells, different fluorescent-labeled anti-CEA antibodies have allowed clear tumor delineation.^18^–^20^ Our study aimed to assess the tolerability and feasibility of intraoperative fluorescence tumor imaging using SGM-101 for patients undergoing a surgical exploration for PDAC.

## Methods

### Study Design

This study was an open-label, single-ascending-dose, exploratory study of 12 patients approved by a certified medical ethics review board (clinicaltrials.gov ID: NCT02973672). The study primarily aimed to assess SGM-101 with respect to tolerability and performance in the intraoperative detection of PDAC lesions.

Due to the exploratory nature of the study, sample size was not based on a formal calculation of statistical power. A dose-escalating scheme with intravenous doses of 5, 7.5 and 10 mg administered 48 or 96 h before surgery was used based on our preclinical data.^18^ We included patients with elevated plasma CEA levels (> 3 ng/mL) who had a clinical diagnosis of PDAC and were scheduled for surgical exploration.

The exclusion criteria ruled out pregnancy, history of allergic reactions, impaired renal function, plasma CEA level higher than 300 ng/mL and a diagnosis of another malignancy within the last 5 years. Tolerability assessment (adverse events, electrocardiogram, blood pressure, pulse, peripheral oxygen saturation, respiratory rate, and temperature), routine laboratory tests, and urine collection for urinalysis were performed at regular intervals, starting just before SGM-101 administration and lasting until 12 h after dosing.

On both the day of surgery and the first postoperative day, measurements were repeated. Follow-up visits coinciding with clinical care took place on the day of discharge and at the first outpatient clinic visit. Adverse events and the concomitant use of other medications were recorded throughout the study period.

All surgical procedures were open procedures, and the surgical field was explored using standard visual and tactile methods, complemented with ultrasound based on the surgeon’s preference. Subsequently, the fluorescence imaging system was used to identify NIR-fluorescent lesions. All lesions identified by visual/tactile methods and/or NIR fluorescence were resected if resection was surgically feasible and clinically relevant. Each resected lesion was marked on a case report form as fluorescent or non-fluorescent and as clinically suspected of malignancy or not.

After resection, fluorescence imaging of the resected specimen (before and after slicing) was performed in the pathology department. The slice containing the peak fluorescence signal was imaged using the Pearl imager and corresponding software (LI-COR, Lincoln, NE, USA). The fluorescence signal in tumor and background tissue (tumor-to-background ratio [TBR]) was quantified using ImageJ (version 1.49b; NIH, Bethesda, MD, USA; http://imagej.nih.gov/ij/) on tagged image file format (TIFF) images (8 bits) subtracted from intraoperative videos. Tumor status was correlated with intraoperative fluorescent assessment. A tumor-positive, fluorescent lesion was regarded as a true-positive. A tumor-negative, fluorescent lesion was regarded as a false-positive, and a tumor-positive, non-fluorescent lesion was regarded as a false-negative.

In addition, immunohistochemistry (IHC) was performed to demonstrate CEA expression. To correlate SGM-101 presence with tumor status and CEA expression on a microscopic level, sections were scanned using the Odyssey imager (LI-COR). In this report, both TBR and patient characteristics are reported as mean ± standard deviation and range.

### Investigational Product

The tumor-specific imaging agent, SGM-101 (molecular weight, 148.6 kDa), consists of an anti-CEA chimeric monoclonal antibody covalently bound to fluorophore BM-104 (excitation, 686 nm; emission, 704 nm). Approximately 30–40% of the antibody is unconjugated, and the average number of fluorophores per antibody is between 1 and 2.

In compliance with good manufacturing practice (GMP), SGM-101 was manufactured by Novasep (Gosselies, Belgium). The agent was supplied by Surgimab (Montpellier, France) as a sterile solution for injection in amber glass vials containing 10 mg of SGM-101 in 10 mL and stored in frozen form at − 20 °C. Before SGM-101 administration, the frozen vials were thawed and diluted in 100 mL of 0.9% NaCl and infused for 30 min.

### Imaging System

Imaging was performed using the Artemis and Spectrum fluorescence imaging systems (Quest Medical Imaging, Middenmeer, the Netherlands^21^). Both systems consist of three wavelength-isolated light sources, including a “white” light source and two separate NIR light sources.

For this study, the Cy5,5 filter setting (fluorescent range, 680 ± 30 nm) was used. Color video and fluorescence images were acquired simultaneously by separate sensors and displayed in real time using custom-built optics and software, thereby displaying color video and NIR fluorescence images separately. A pseudo-colored (lime green) merged image of the color video and fluorescence images also was generated. The gain and exposure time settings were controlled using the Quest software. An average gain setting of 25 was used, and exposure time was varied between 60 and 120 ms according to the clinical situation. The camera was attached to a freely moveable arm. During surgery, the camera and moveable arm were enclosed in a sterile shield and drape (Medical Technique Inc., Tucson, AZ, USA).

## Results

### General

The study investigated 12 patients. Demographics and details on surgical procedure are summarized in Table 1.

**Table 1:** Demographics and details of SGM-101 dosing, surgical procedure, fluorescence imaging, and correlation with histopathology

Demographics				SGM-101 dosing		Surgical procedure	Fluorescence imaging and correlation with histopathology										
							Primary tumor						Metastases				
ID no.	Age (years)	Sex	CEA serum (ng/mL)	Dose (mg)	Timing (days preop)	Procedure	Fluorescence primary tumor	TBR	Resection	TBR Pearl	Histopathology	Intensity CEA staining	Fluorescence metastasis	Location	TBR	Histopathology	Intensity CEA staining
201	71	M	10.6	5	2	Abandoned, metastases	Yes	1.6	No	–	–	–	Yes	Liver	1.4	Adenocarcinoma	Moderate
														Peritoneum	1.8	Adenocarcinoma	Moderate
202	61	F	44.2	5	2	Abandoned, unresectable	Yes	1.6	No	–	–	–	No	–	–	–	–
203	66	M	3.4	5	2	Whipple, R0	Yes	1.4	Yes	4.1	Adenocarcinoma	Weak	No	–	–	–	–
204	67	F	3.5	7.5	2	Abandoned, unresectable	Yes	1.4	No				No	–	–	–	–
205	52	M	5.7	7.5	2	PPPD, R0	Yes	1.6	Yes	1.7	IPMN	Moderate	No	–	–	–	–
206	68	M	23.5	7.5	2	Abandoned, metastases	–	–	No	–	–	–	Yes	Liver (segment 2)	1.2	Adenocarcinoma	Strong
														Liver (segment 3)	1.4	Adenocarcinoma	Strong
207	80	M	4.5	7.5	4	Whipple, R0	Yes	1.4	Yes	3.1	Adenocarcinoma	Moderate	No	–	–	–	–
208	70	M	4.9	7.5	4	PPPD, R1	Yes	1.3	Yes	3.3	Adenocarcinoma	Strong	No	–	–	–	–
209	71	M	41.1	7.5	4	Abandoned, metastases	Yes	2.3	No	–	–	–	Yes	Liver^a^	2.2	Adenocarcinoma	Strong
210	66	F	2.4	10	4	PPPD, R0	Yes	1.5	Yes	2.7	Adenocarcinoma	Strong	No	–	–	–	–
211	65	F	34.4	10	4	Total pancreatectomy, R0	Yes (head)	1.4	Yes	3.8	Adenocarcinoma	Strong	No	–	–	–	–
							Yes (tail)^b^	1.4	Yes	3.4	Adenocarcinoma	Strong					
212	70	M	2.8	10	4	Abandoned, unresectable	Yes	1.7	No	–	–	–	No	–	–	–	–

*CEA* carcinoembryonic antigen, *TBR* tumor-to-background ratio, *M* male, *F* female, *PPPD* pylorus-preserving pancreatico duodenectomy, *IPMN* intraductal papillary mucinous neoplasm

^a^A possible liver metastases (3 mm) was also seen on computed tomography (CT) and magnetic resonance imaging (MRI)

^b^Second primary tumor

### Dose Escalation

**Fig. 1:**
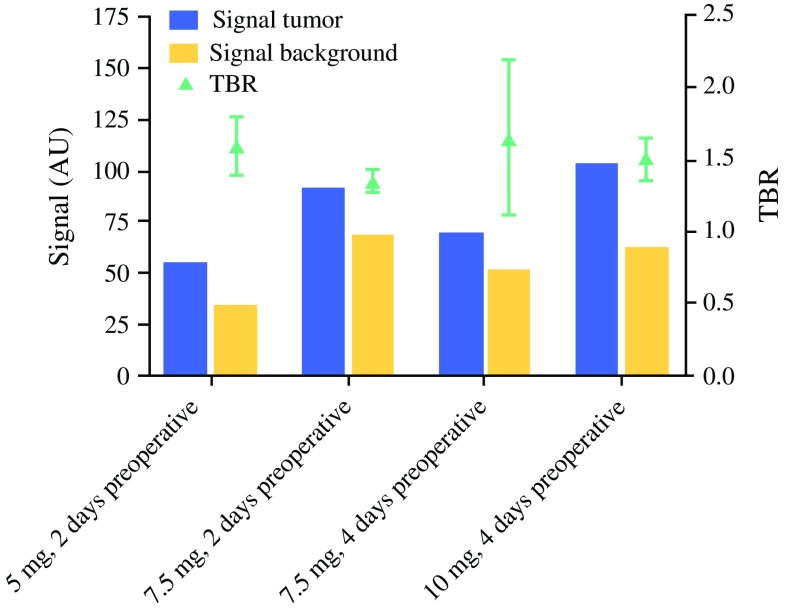
Tumor and background fluorescence signal (in arbitrary units [AU]) and mean tumor-to-background ratio (TBR) per dose group. The influence of SGM-101 dose and timing on the fluorescence signal and TBR seemed limited in this study

Because the increase from 5 to 7.5 mg did not cause an increase in TBR, the interval between SGM-101 administration and surgery was prolonged to 96 h, while the dose remained equal at 7.5 mg for the following cohort. The longer interval between SGM-101 administration and surgery resulted in an improvement of TBR. Hence, the last dose level was increased to 10 mg, while the longer interval was retained. The TBR results are summarized in Fig. 1.

### Safety Data

One patient (ID no. 208) experienced mild, self-limiting abdominal discomfort and diarrhea on the day of SGM-101 administration (dose, 7.5 mg), although these symptoms likely were not related to SGM-101. Causality could not be excluded. In the remaining patients, adverse events related to SGM-101 were not seen. After the surgical procedure, four severe adverse events were noted, all related to the surgical procedure or to disease progression.

### Primary Tumor

**Fig. 2:**
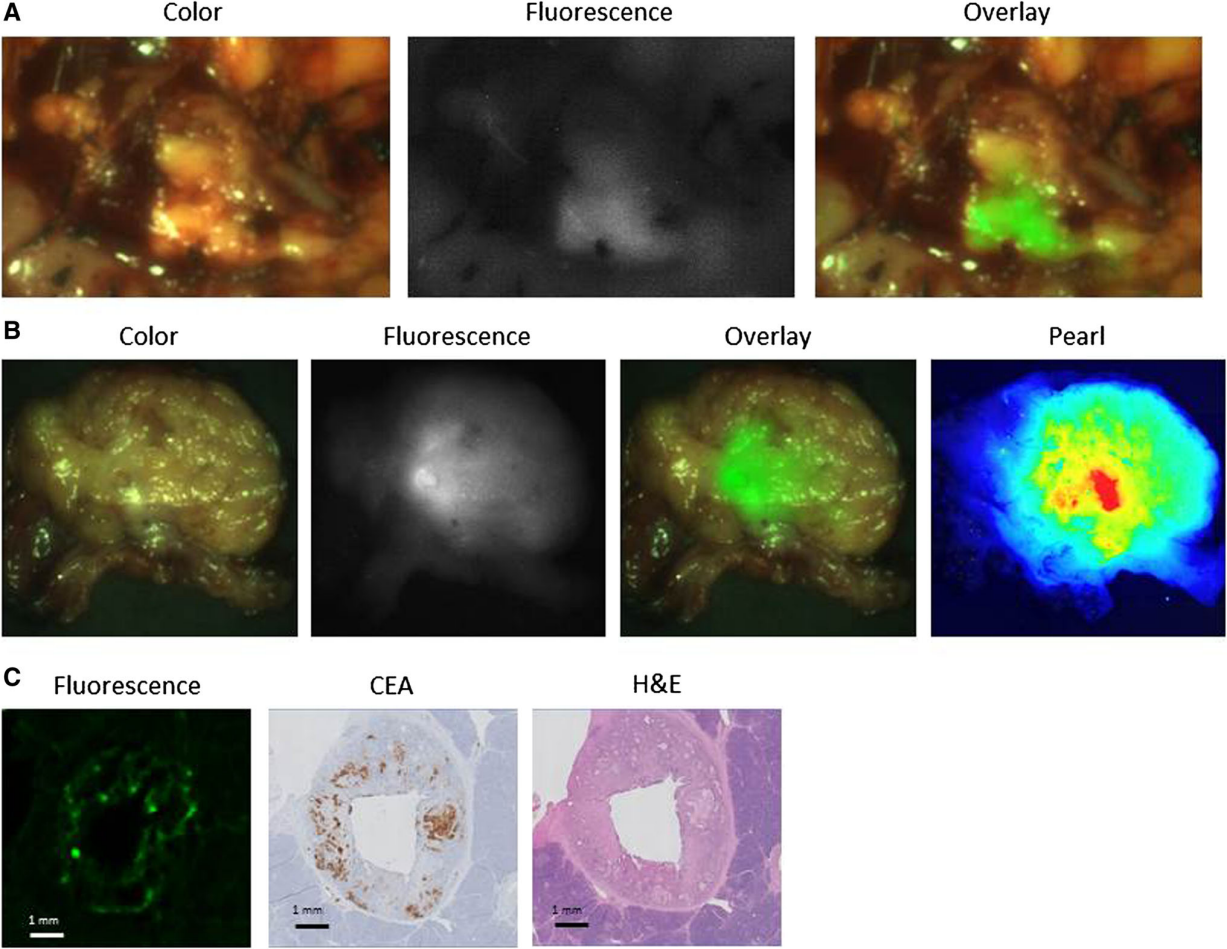
Fluorescence detection of a primary pancreatic tumor. **a** Color (left column), fluorescence (middle column), and merged (right column) images from intraoperative imaging of a pancreatic tumor using the Quest imaging system. **b** Color (left column), fluorescence (middle left column), and merged (middle right column) images of ex vivo imaging of a slice from the same pancreatic tumor using the Quest imaging system and to the Pearl imager (right column). **c** Histopathologic evaluation and fluorescence signal in a primary pancreatic tumor. Fluorescence microscopy (left column) showing accumulation of SGM-101 in tumor cells. The fluorescence pattern is consistent with carcinoembryonic antigen (CEA) expression measured using immunohistochemistry (IHC, middle column), which corresponds to the site containing tumor cells visible on hematoxylin and eosin (H&E) staining (right column). *Note:* The acuity of the images is suboptimal compared with the intraoperative setting because these tagged image file format (TIFF) images (8 bits) were subtracted from the intraoperative videos

In one patient (ID no. 206), the surgical procedure was abandoned before the primary tumor was visualized after detection of occult liver metastases. In the remaining 11 patients, the primary tumor was always visualized with fluorescence imaging (TBR, 1.6 ± 0.37; range, 1.3–2.3; Fig. 2 and Table 1). Tumor specimens were not available if the surgical procedure was abandoned due to unresectability or metastases. Consequently, ex vivo fluorescence measurements and correlation of fluorescence signal and histopathology were assessed in seven primary tumors. Assessment of one slice containing peak fluorescence per resection specimen with the Pearl demonstrated a mean TBR of 3.2 ± 0.79 (range, 1.7–4.1; Table 1). Six primary tumors were confirmed as adenocarcinomas on histopathology. The IHC for CEA showed moderate to strong CEA overexpression (Table 1). Odyssey scans of the fluorescence signal in the primary tumor sections showed concordance with tumor cells on histopathology (Fig. 2). The remaining tumor was an intraductal papillary mucinous neoplasm (IPMN) with low-grade dysplasia. Because this is a pre-malignant condition, the fluorescence signal was deemed false-positive. Moderate CEA expression was present in 10–50% of IPMN lesional cells, explaining the fluorescence.

### Other Lesions

**Fig. 3:**
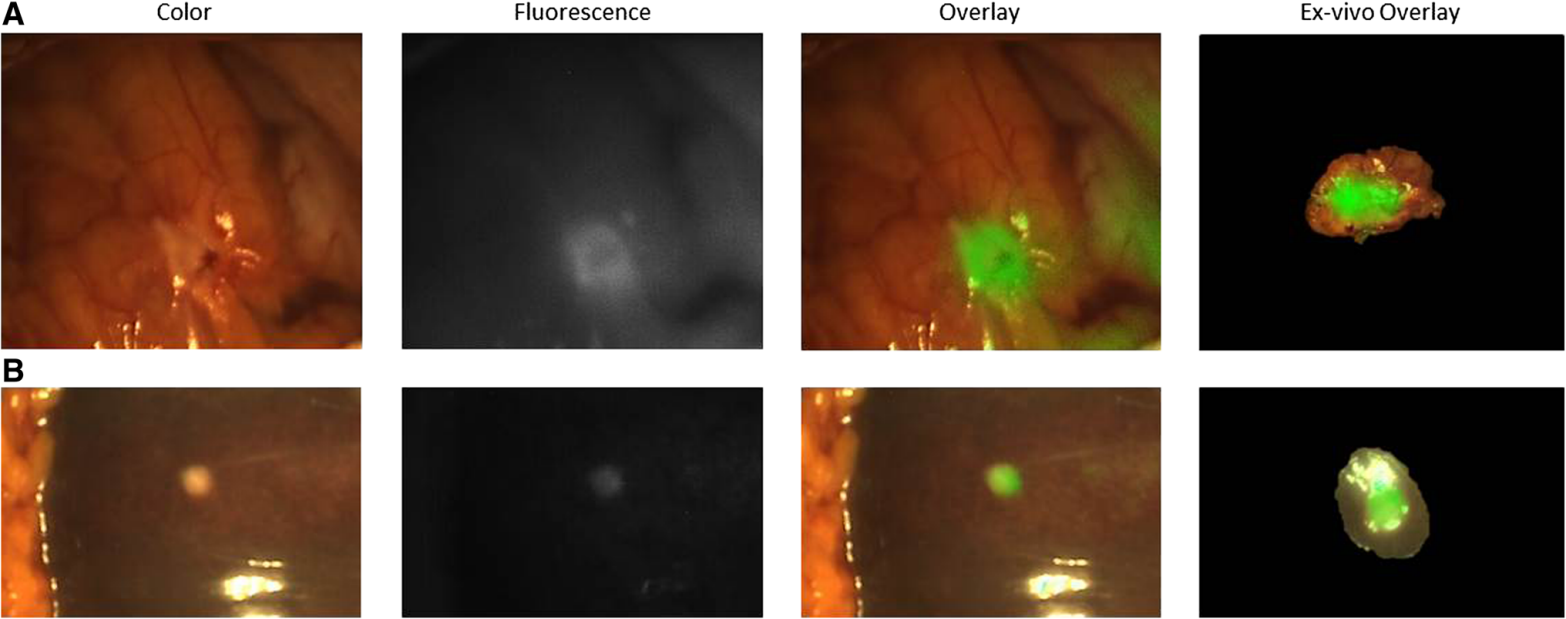
Fluorescence detection of a peritoneal and liver metastasis of a pancreatic tumor. **a** Color (left column), fluorescence (middle left column), and merged (middle right column) images of intraoperative imaging showing a peritoneal metastasis of a pancreatic tumor and images of ex vivo imaging showing a slice from the same metastasis (right column) using the Quest imaging system. **b** Color (left column), fluorescence (middle left column), and merged (middle right column) images of intraoperative imaging showing a liver metastasis of a pancreatic tumor and images of ex vivo imaging showing a slice from the same metastasis (right column) using the Quest imaging system. *Note:* The acuity of the images is suboptimal compared with the intraoperative setting because these tagged image file format (TIFF) images (8 bits) were subtracted from the intraoperative videos

In three patients, liver and/or peritoneal metastases were identified (with both methods) during surgery, whereas in only one patient (ID no. 209), a possible liver metastasis (3 mm) was seen on cross-sectional imaging (computed tomography [CT] scan). All metastatic lesions were fluorescent (TBR, 1.7 ± 0.42; range, 1.2–2.3; Fig. 3). The IHC for CEA showed moderate to strong CEA overexpression (Table 1). A total of eight clinically suspect, non-fluorescent lesions were resected. In two patients. This non-fluorescent tissue contained malignancy, a suspected tumor ingrowth in the common hepatic artery (TBR, 1.3; ID no. 204), and a suspected 1-cm aorta-caval lymph node (TBR, 1.1; ID no. 207).

In frozen-section analysis, the biopsy of the ingrowth in the common hepatic artery demonstrated adenocarcinoma (i.e., false–negative). Frozen section analysis precluded further ex vivo assessment of this biopsy material. The non-fluorescent lymph node contained a metastasis and also was considered a false-negative. Remarkably, ex vivo imaging using the Pearl demonstrated localization of fluorescence, and IHC staining demonstrated CEA expression.

## Discussion

We assessed the safety and feasibility of intraoperative fluorescence imaging of PDAC using SGM-101 in 12 patients. Administration of SGM-101 doses of 5–10 mg appeared to be safe because the occurrence of a possibly related adverse event was limited to one subject. Moreover, administration of SGM-101 did not cause changes in safety measurements including vital signs, ECG, and routine chemistry, hematology, and coagulation laboratory tests.

Intraoperative fluorescence imaging of PDAC was feasible because fluorescence could be detected in primary tumors as well as liver and peritoneal metastases. This demonstrates that despite the suboptimal intrinsic characteristics of PDAC, including poor vascularization, SGM-101 can reach and bind the CEA-expressing tumor cells. However, the TBRs in this study were more modest than for other tumor types (1.6 for primary tumors and 1.7 for metastases). This also could be explained by the histopathology of pancreatic adenocarcinoma, with solitary ducts of tumor cells in preexistent normal pancreatic tissue and a remarkable desmoplastic stroma, which could make the fluorescence pattern more sparse than with other tumor types. Finally, SGM-101 fluoresces at about 700 nm, and this “far red” part of the spectrum is associated with more autofluorescence of the surrounding background.

Overlying tissue and blood in the surgical field likely caused both cases of intraoperative false-negativity in this study. The maximal depth of fluorescence signal penetration is approximately 1 cm below the tissue surface.^22^ Consequently, more deeply seated tumors will not be detectable using fluorescence imaging. Moreover, absorption of the fluorescence signal by blood can have a negative impact on the detection of the fluorescence signal.^23^ Thus, it may be beneficial to combine fluorescence with other methods, such as radionuclides or photo-acoustic imaging, to increase detection of deeply seated or covered tumors. For example, a study with administration of (111)In-girentuximab-IRDye800CW has been initiated for patients with clear cell renal cell carcinoma. An ex vivo study with this agent has already demonstrated that uptake in tumor tissue can be visualized using both radionuclide and fluorescence imaging.^24^ The advantage of a radionuclide is not only that it allows increased penetration depth, but also that it also allows preoperative scanning.

Based on both preclinical and clinical studies, CEA was chosen as a suitable target for fluorescence imaging of PDAC. The current study confirmed its potential as a target for intraoperative fluorescence imaging. Because PDAC is characterized by several genetic mutations such as KRAS (90%), CDK2NA (90%), TP53 (75–90%), and SMAD4/DPDAC4 (50%),^25^ various other targets are expressed on PDAC including integrin αvβ6, EGFR, and uPAR.^10^ Once results from other clinical studies using different tumor-specific agents become available, a cocktail of selected agents likely will be used in a personalized manner to increase the yield of fluorescence imaging.^5^ In addition, the targeting of tumor stroma could be pursued to increase sensitivity. Because PDAC is composed of abundant desmoplastic stroma located at the invasive front of the tumor, this tumor is particularly suited for stroma targeting.^26^

By demonstrating that both primary tumors and small metastases can be visualized using intraoperative fluorescence imaging, this study provided the first step toward implementing fluorescence-guided PDAC surgery. However, larger clinical studies are needed to assess whether this technique allows evaluation of resectability and margin assessment (including vascular involvement) and whether this will ultimately translate into improved overall survival.

Moreover, several challenges need to be addressed before fluorescence imaging can be implemented in a broader surgical practice. Funding and awareness are required to initiate phase 3 multicenter trials. Regulatory hurdles for approval of both imaging agents and imaging systems need to be overcome, and standardization of imaging systems is required to ensure accurate and reproducible results.^27^

To the best of our knowledge, this study is the first to describe a tumor-specific imaging agent for intraoperative fluorescence imaging of PDAC. The use of a fluorescent-labeled anti-CEA antibody was safe and feasible for the detection of both primary tumors and metastases. These results underscore the great potential of image-guided surgery, but more prospective research is necessary to establish the effect on clinical decision making and overall survival.
